# Supplementary figures and images for: Discussion on the relationship between gut microbiota and glioma through Mendelian randomization test based on the brain gut axis
Source: PLoS One. 2024 May 29;19(5):e0304403. doi: 10.1371/journal.pone.0304403 (PMC11135782; doi:10.1371/journal.pone.0304403)

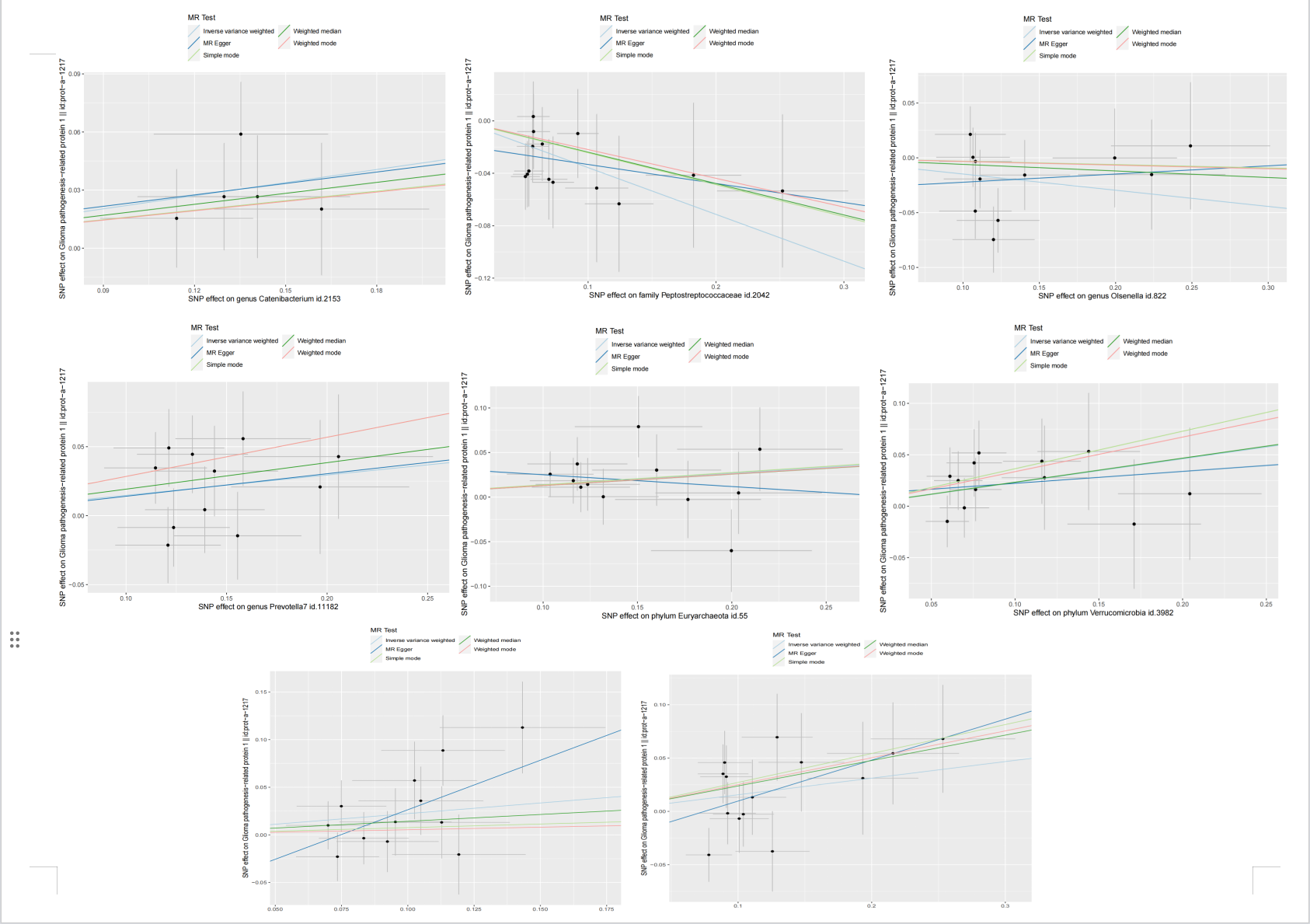

Supplement: S1 Fig — (PNG) [file pone.0304403.s001.png]

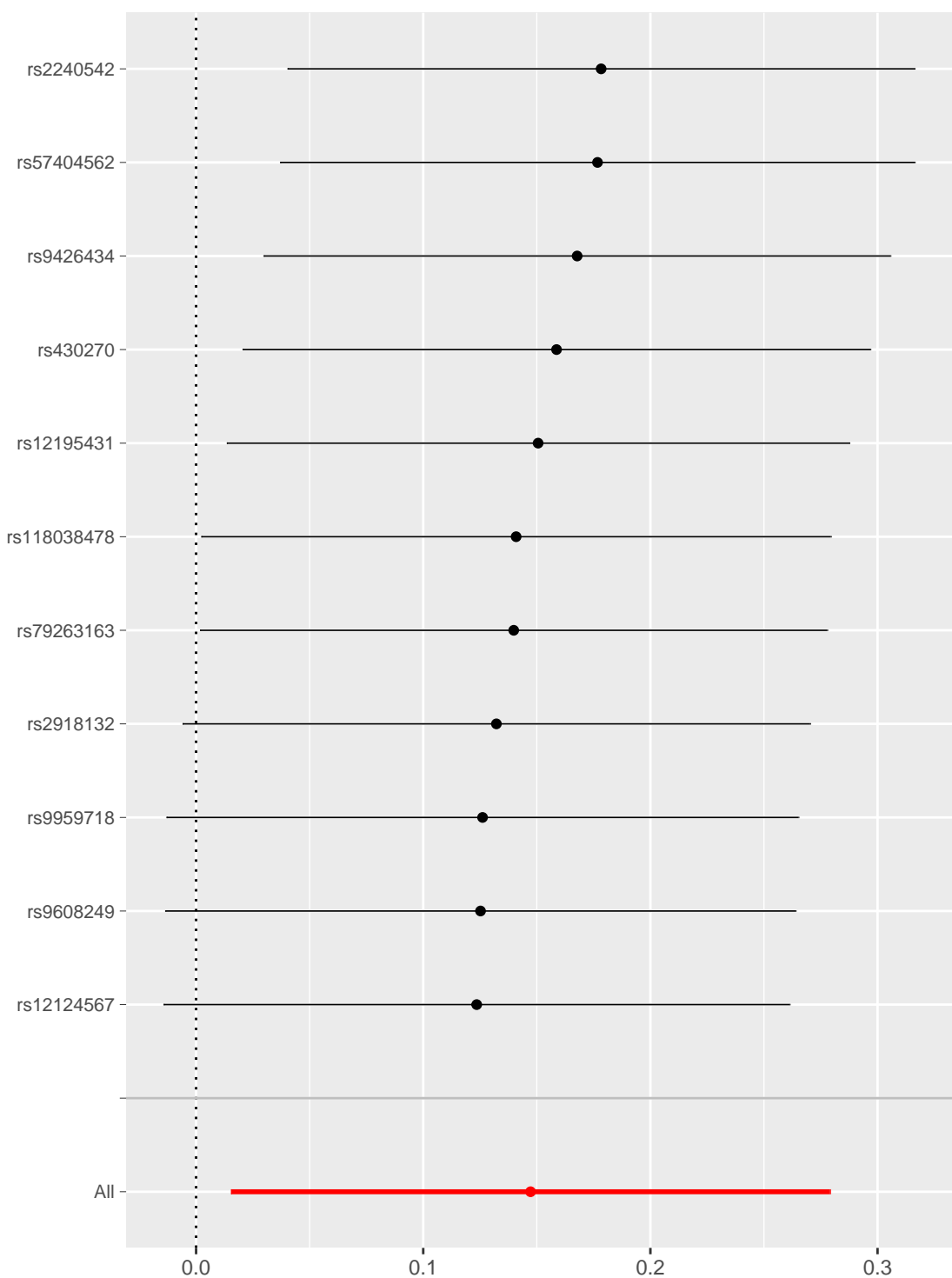

Supplement: S2 Appendix — (PDF) [file pone.0304403.s006.pdf]

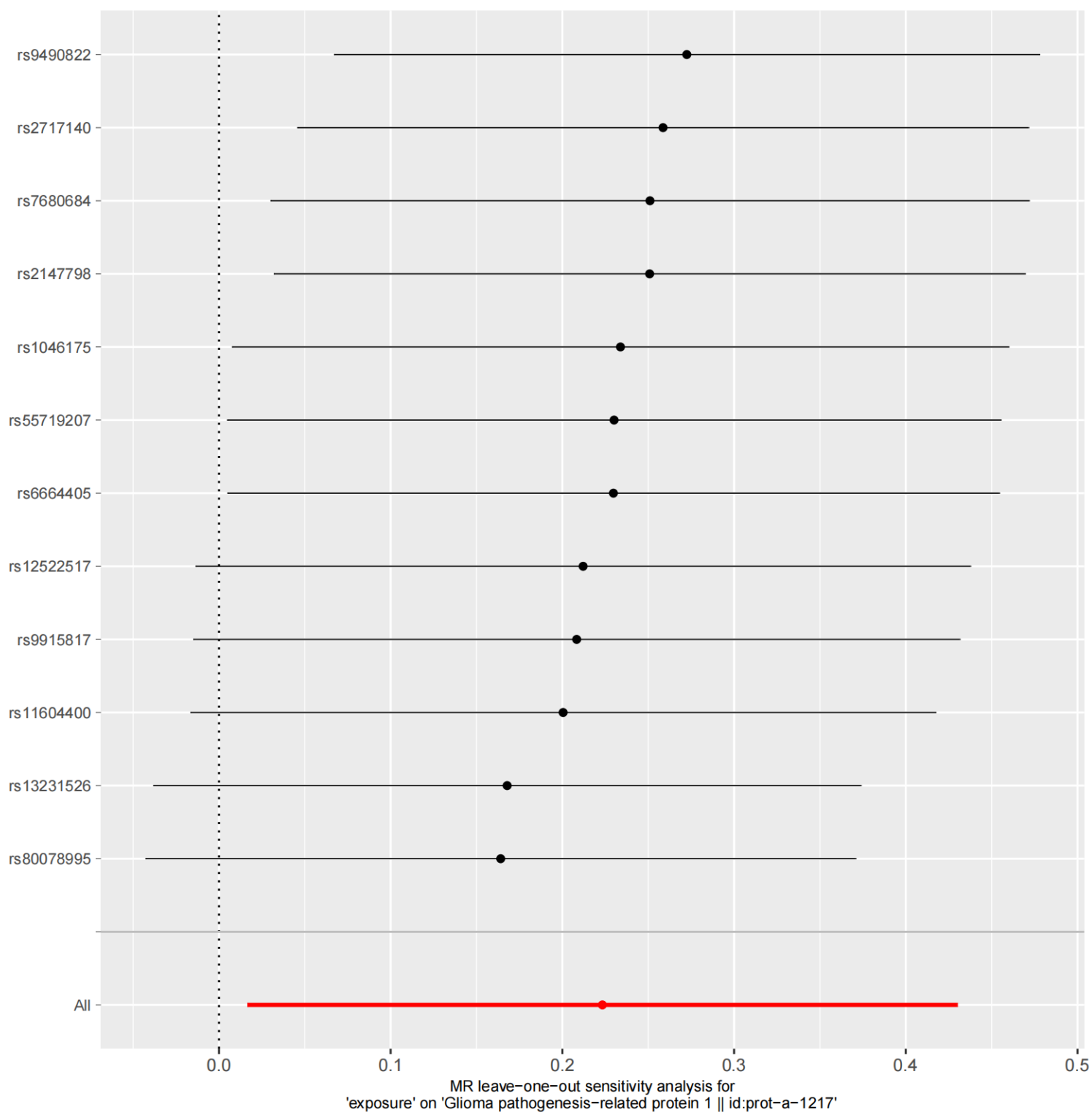

Supplement: S3 Appendix — (PDF) [file pone.0304403.s007.pdf]

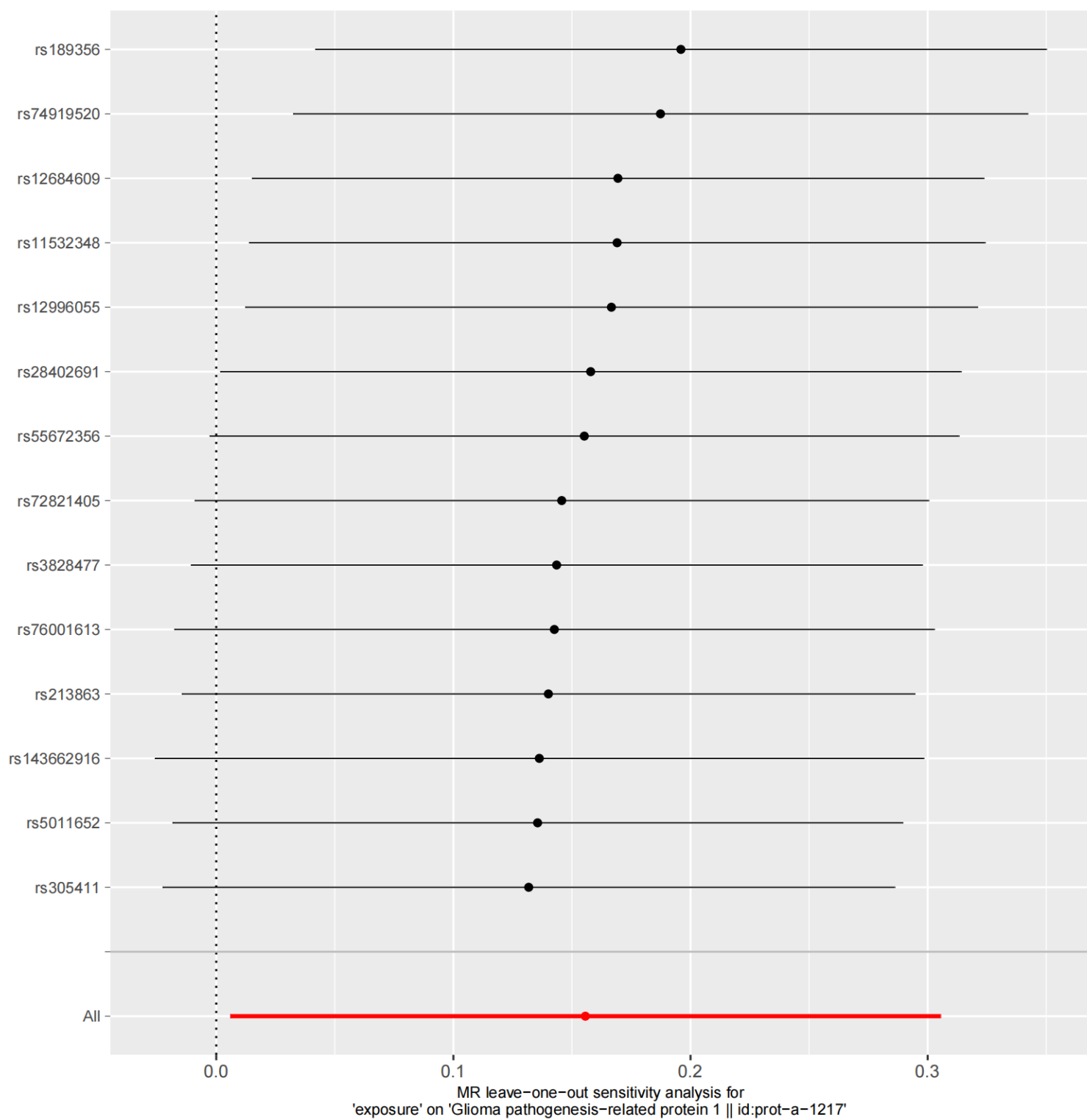

Supplement: S5 Appendix — (PDF) [file pone.0304403.s009.pdf]

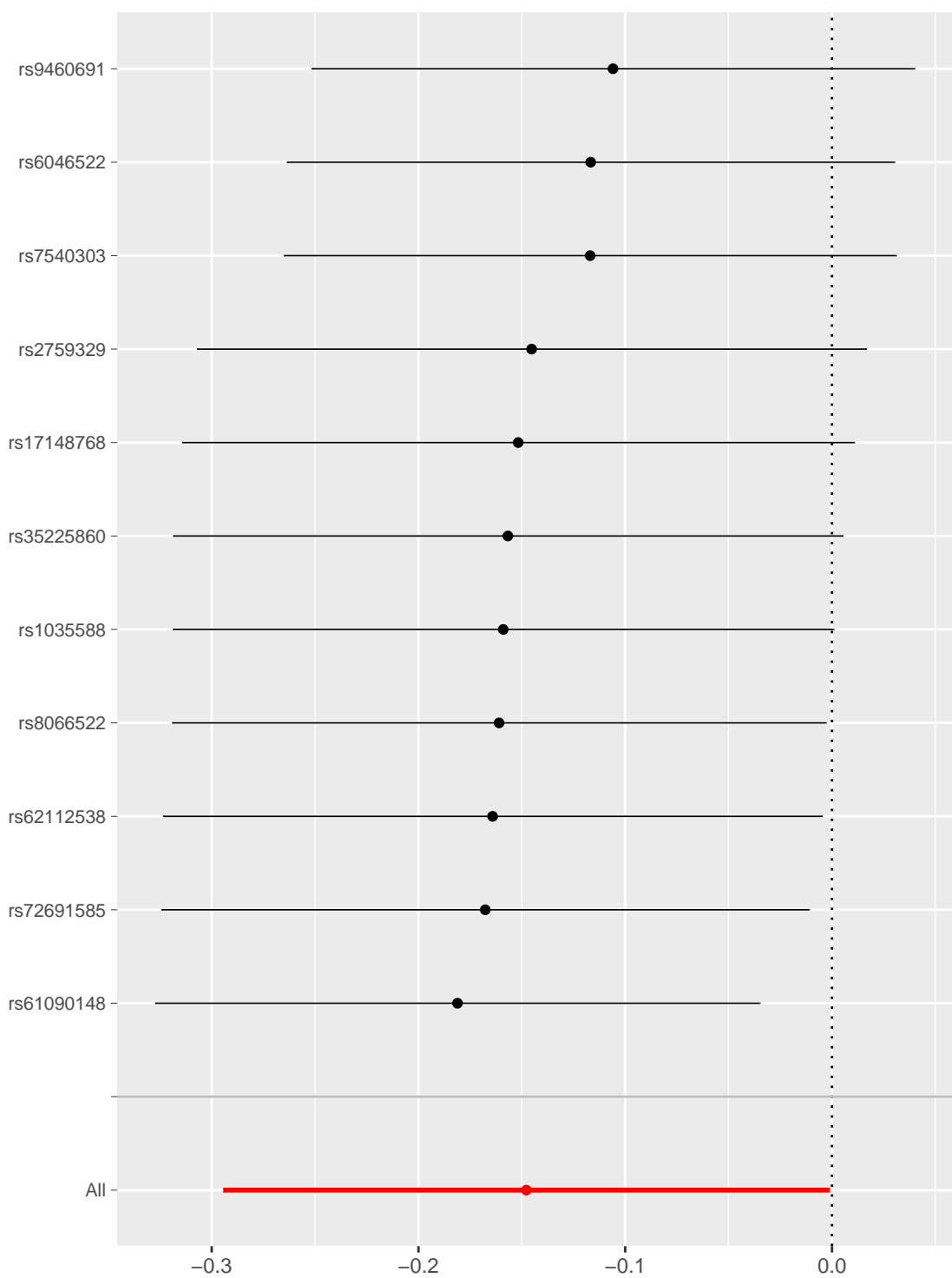

Supplement: S6 Appendix — (PDF) [file pone.0304403.s010.pdf]

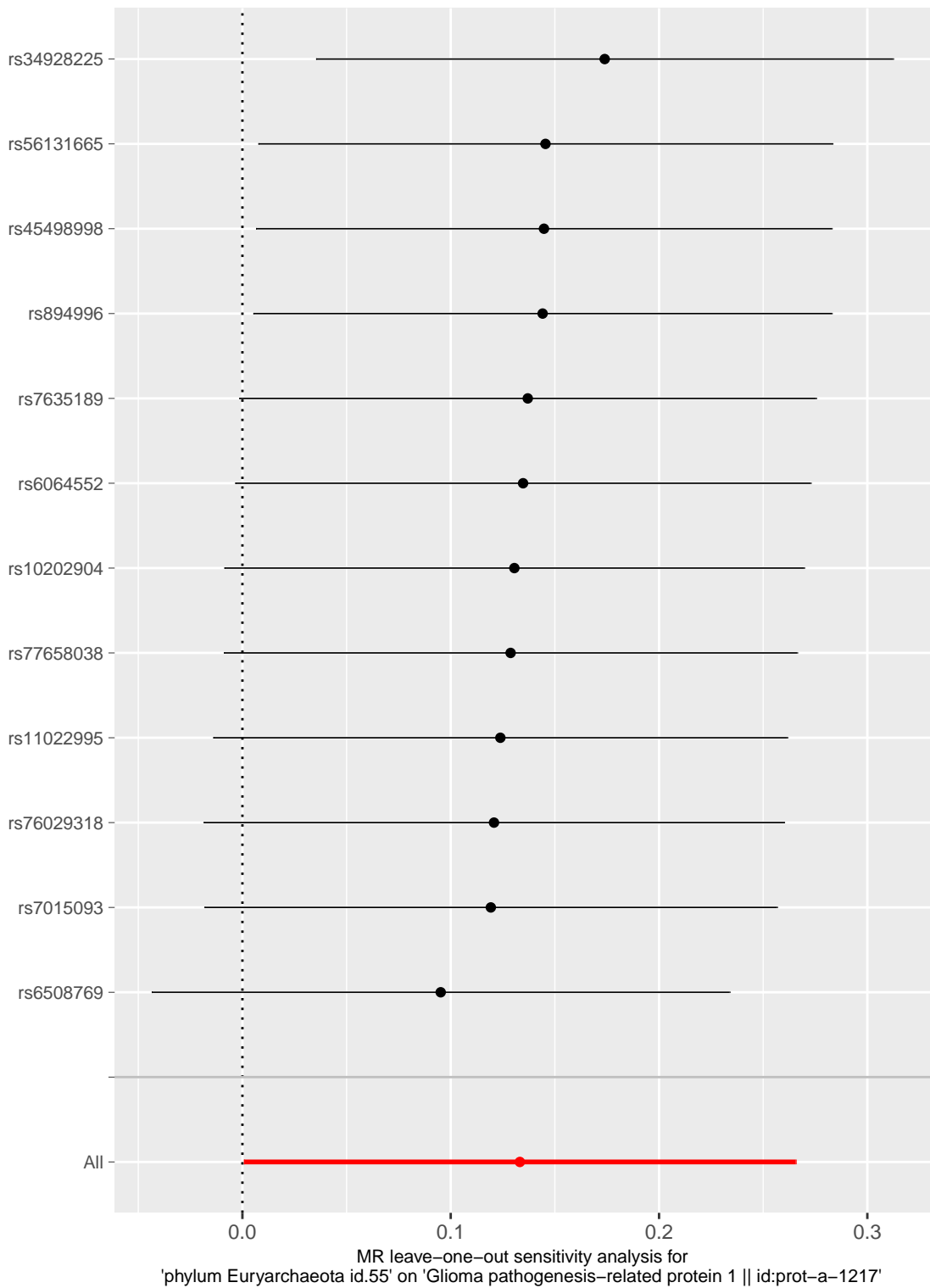

Supplement: S7 Appendix — (PDF) [file pone.0304403.s011.pdf]

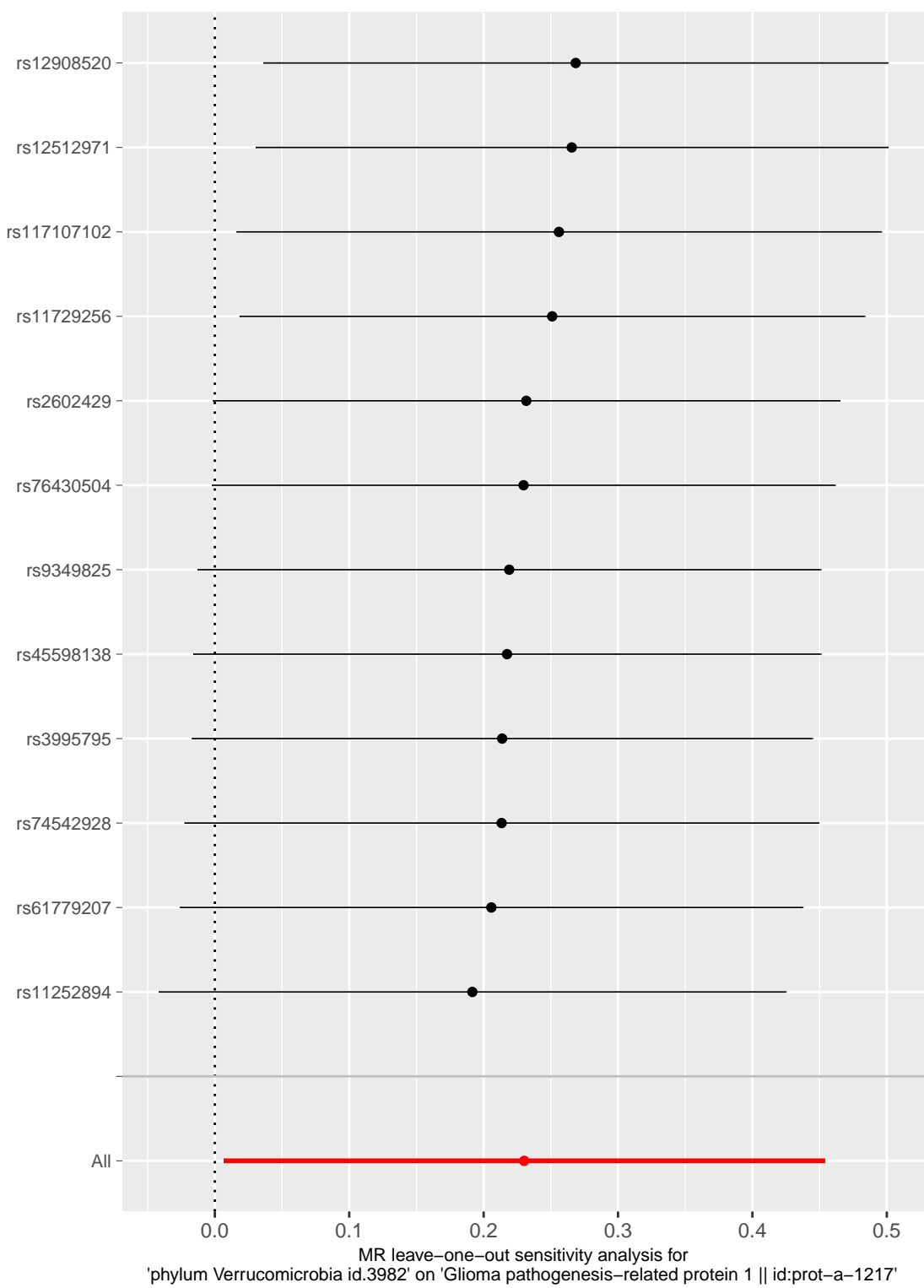

Supplement: S8 Appendix — (PDF) [file pone.0304403.s012.pdf]

SNP effect on Glioma pathogenesis-related protein 1 || id:prot-a-1217

### MR Test

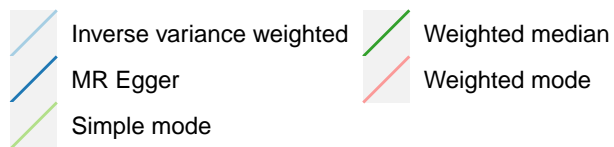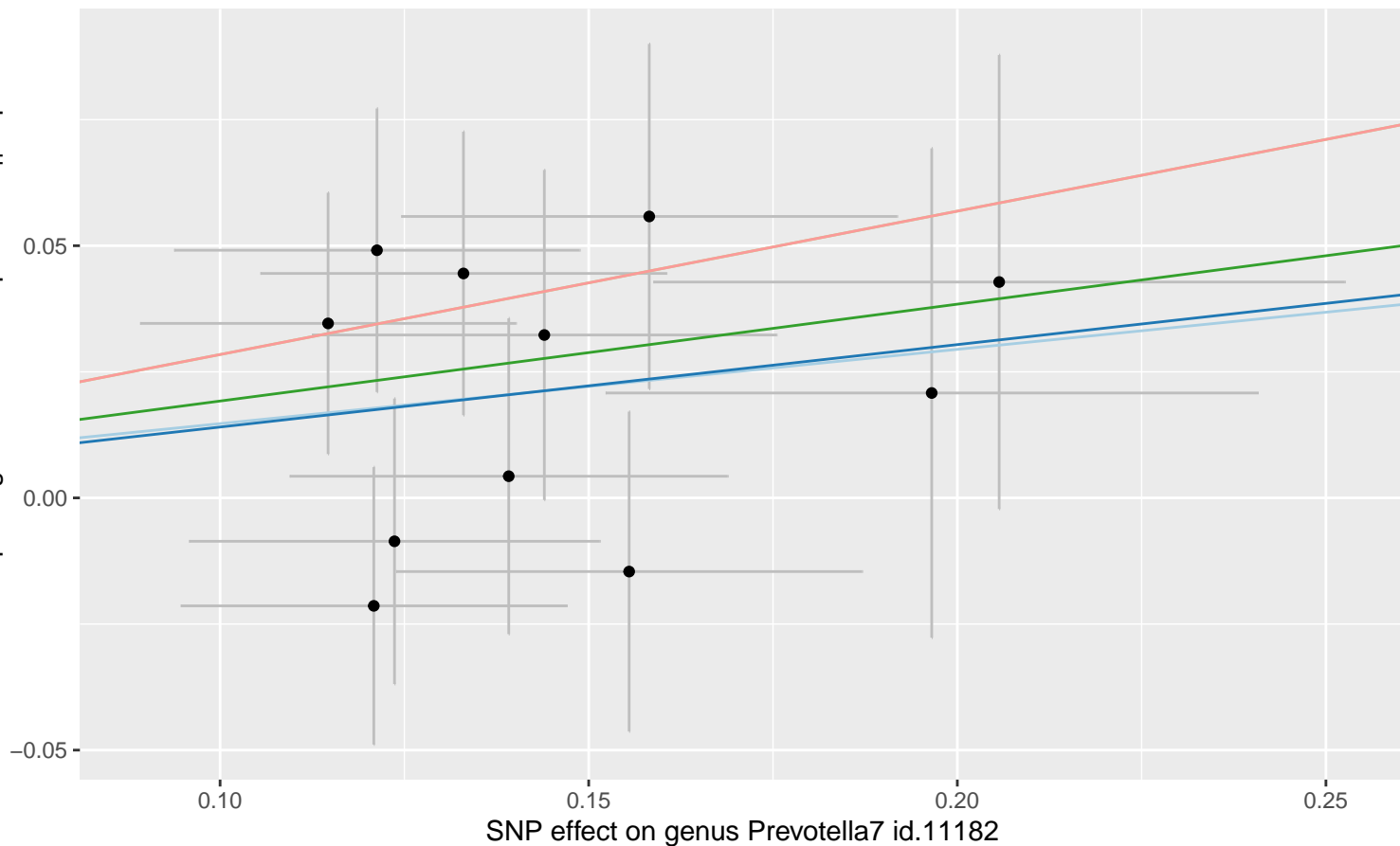

Supplement: S10 Appendix — (PDF) [file pone.0304403.s014.pdf]

# MR Test

- Inverse variance weighted
- MR Egger
- Simple mode
- Weighted median
- Weighted mode

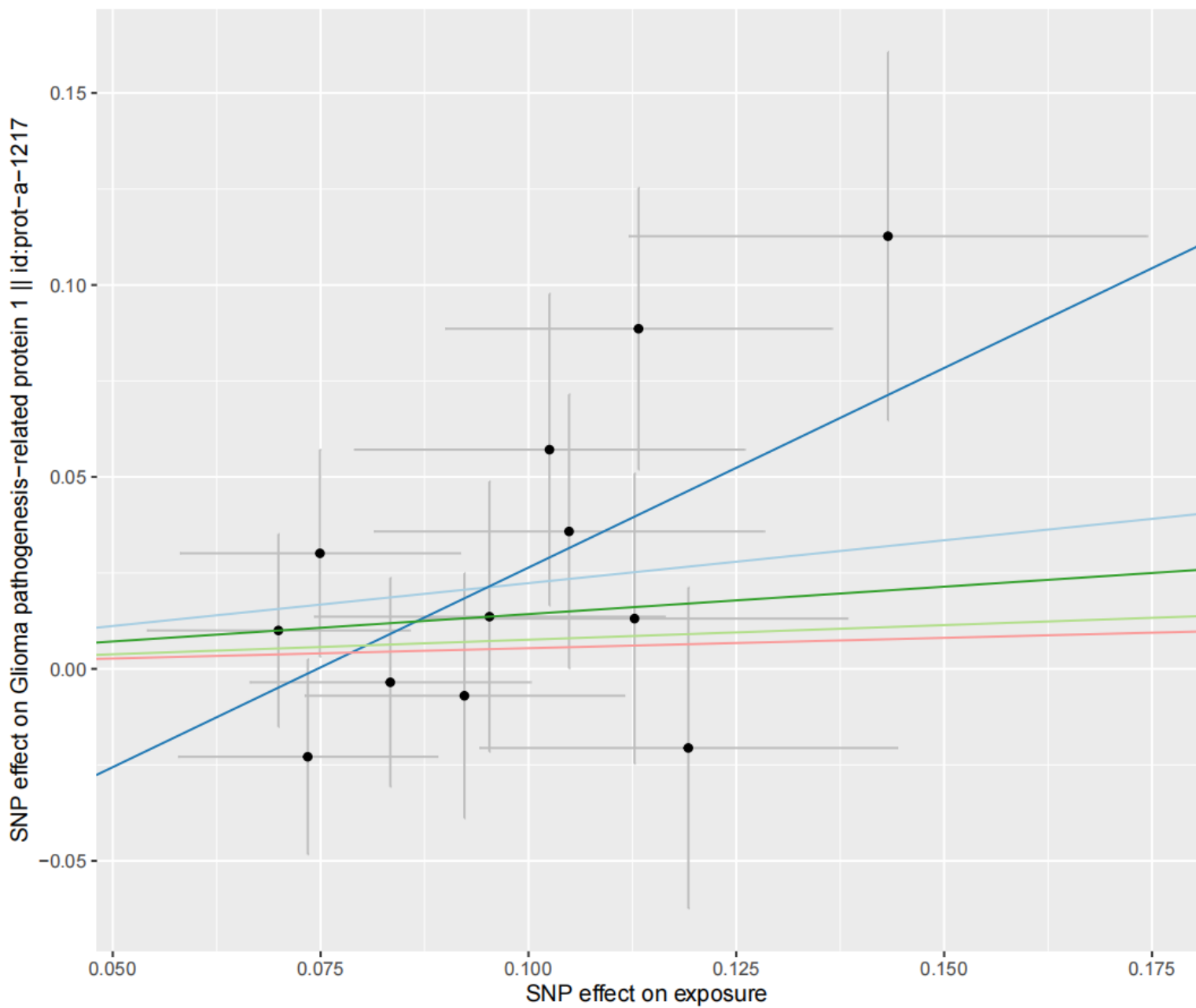

Supplement: S11 Appendix — (PDF) [file pone.0304403.s015.pdf]

SNP effect on Glioma pathogenesis-related protein 1 || id:prot-a-1217

# MR Test

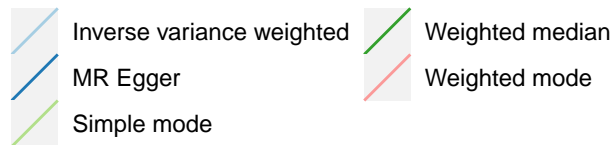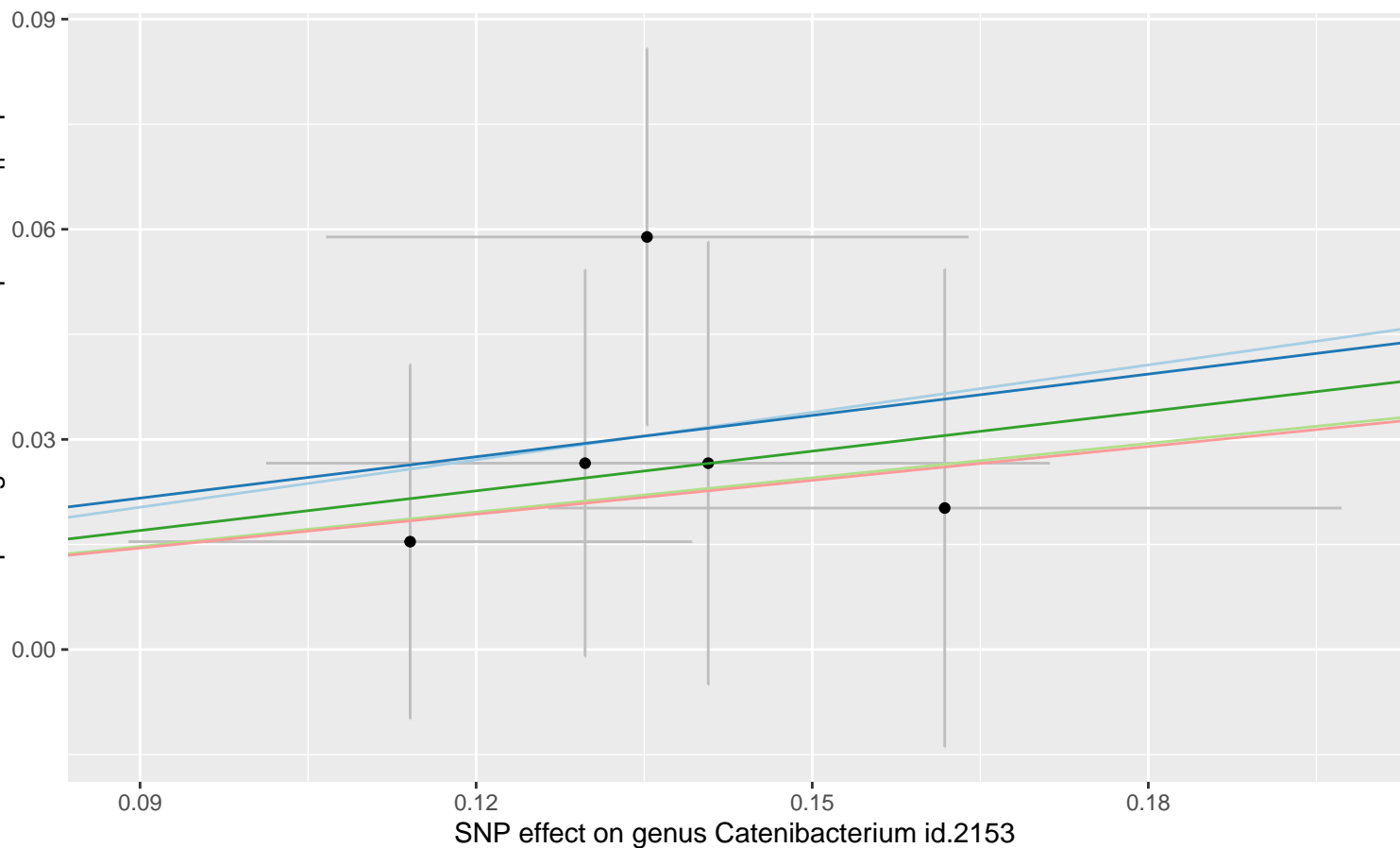

Supplement: S12 Appendix — (PDF) [file pone.0304403.s016.pdf]
