# Supplementary material for: Discussion on the relationship between gut microbiota and glioma through Mendelian randomization test based on the brain gut axis
Source: PLoS One. 2024 May 29;19(5):e0304403. doi: 10.1371/journal.pone.0304403 (PMC11135782; doi:10.1371/journal.pone.0304403)
Supplement: S1 Appendix — (PDF) [file pone.0304403.s005.pdf]

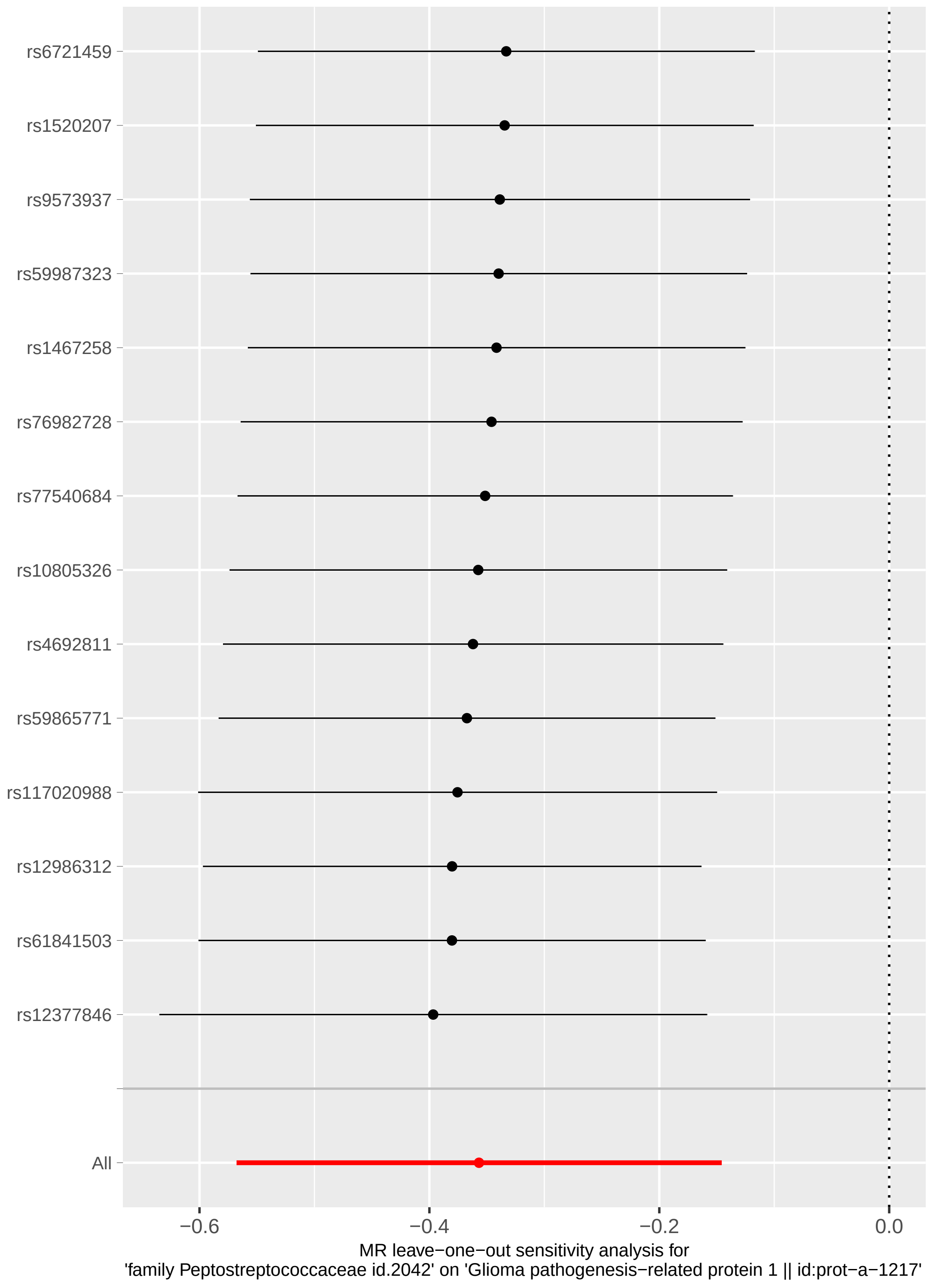
rs6721459
rs1520207
rs9573937
rs59987323
rs1467258
rs76982728
rs77540684
rs10805326
rs4692811
rs59865771
rs117020988
rs12986312
rs61841503
rs12377846
All
−0.6
−0.4
−0.2
0.0
MR leave−one−out sensitivity analysis for
'family Peptostreptococcaceae id.2042' on 'Glioma pathogenesis−related protein 1 || id:prot−a−1217'
