# Supplementary material for: Discussion on the relationship between gut microbiota and glioma through Mendelian randomization test based on the brain gut axis
Source: PLoS One. 2024 May 29;19(5):e0304403. doi: 10.1371/journal.pone.0304403 (PMC11135782; doi:10.1371/journal.pone.0304403)
Supplement: S4 Appendix — (PDF) [file pone.0304403.s008.pdf]

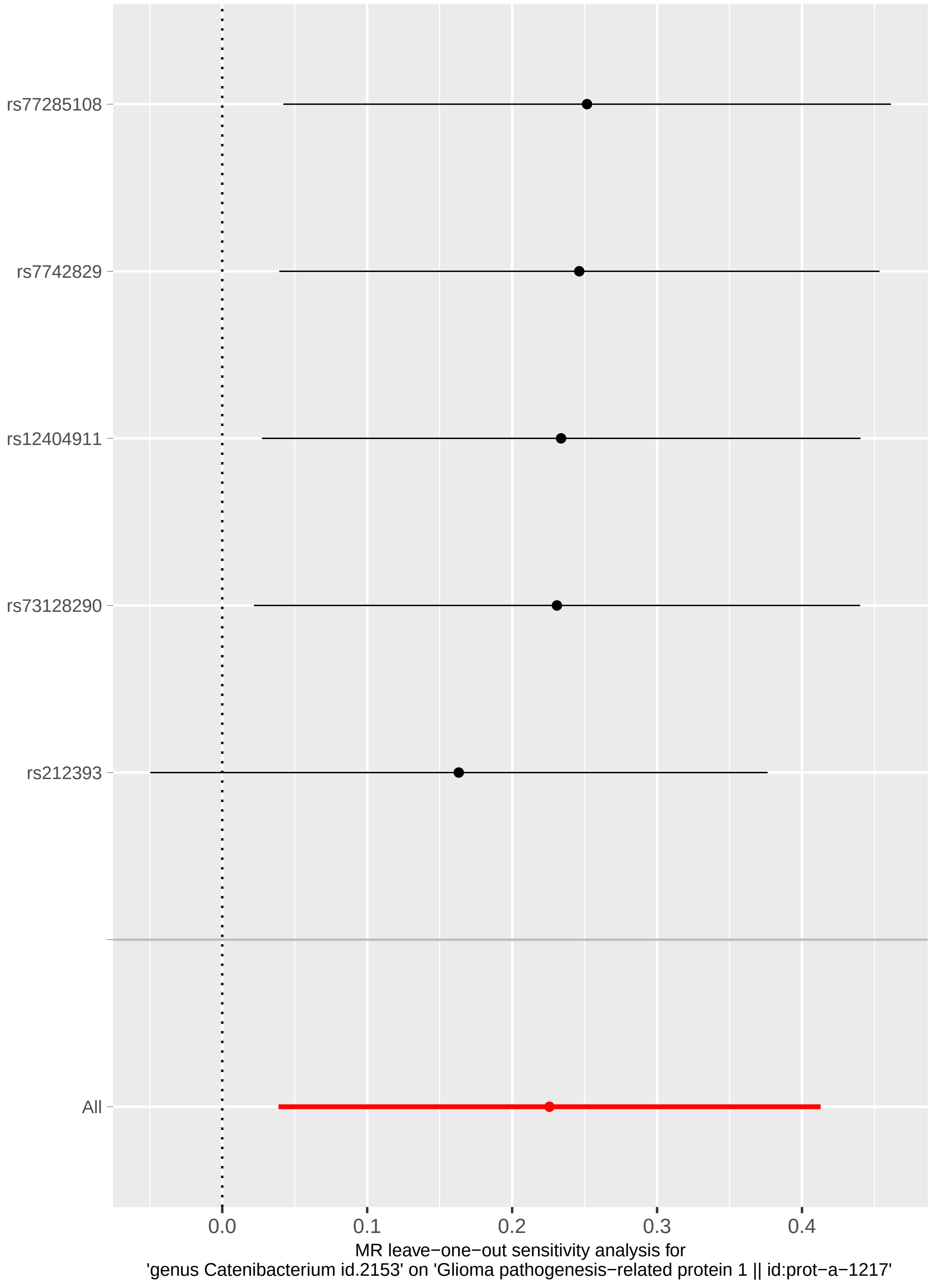
rs77285108
rs7742829
rs12404911
rs73128290
rs212393
All
0.0
0.1
0.2
0.3
0.4
MR leave−one−out sensitivity analysis for
'genus Catenibacterium id.2153' on 'Glioma pathogenesis−related protein 1 || id:prot−a−1217'
