# Supplementary material for: Discussion on the relationship between gut microbiota and glioma through Mendelian randomization test based on the brain gut axis
Source: PLoS One. 2024 May 29;19(5):e0304403. doi: 10.1371/journal.pone.0304403 (PMC11135782; doi:10.1371/journal.pone.0304403)
Supplement: S16 Appendix — (PDF) [file pone.0304403.s020.pdf]

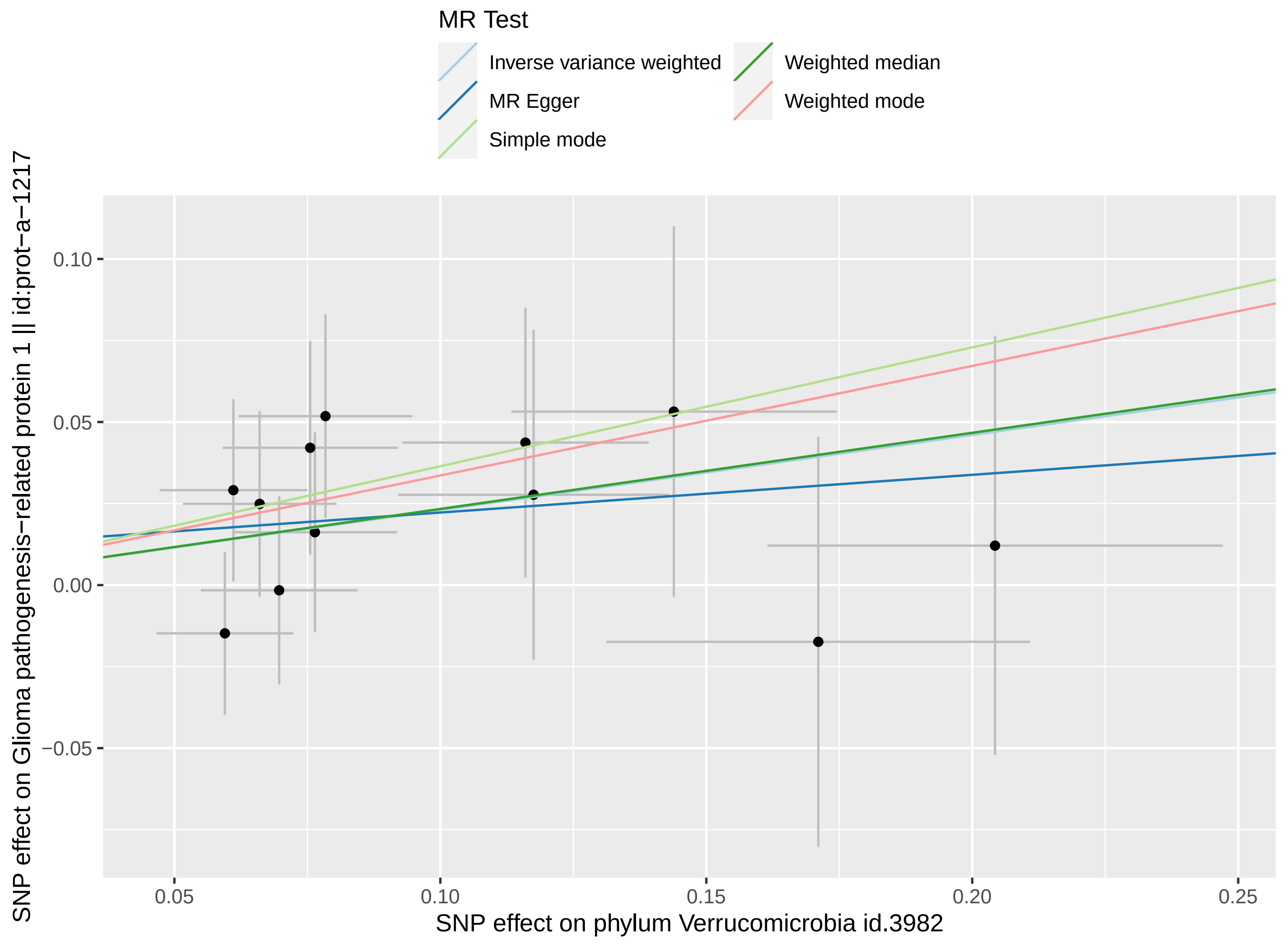
MR Test
Inverse variance weighted
MR Egger
Simple mode
Weighted median
Weighted mode
SNP effect on Glioma pathogenesis−related protein 1 || id:prot−a−1217
0.10
0.05
0.00
−0.05
0.05
0.10
0.15
0.20
0.25
SNP effect on phylum Verrucomicrobia id.3982
