# Supplementary material for: Discussion on the relationship between gut microbiota and glioma through Mendelian randomization test based on the brain gut axis
Source: PLoS One. 2024 May 29;19(5):e0304403. doi: 10.1371/journal.pone.0304403 (PMC11135782; doi:10.1371/journal.pone.0304403)
Supplement: Attachment — Submitted filename: Respond Editor and Reviewers1.docx [file pone.0304403.s021.docx]

Dear Editor and Reviewers,

I am writing this letter to sincerely thank you for taking the time and effort to review our manuscript titled "[Manuscript Title]". Your insightful comments and suggestions have greatly enhanced the quality and coherence of our work. We have carefully considered each of your suggestions and have made corresponding revisions to the manuscript. We believe that these adjustments have significantly improved our paper, making the arguments clearer and more compelling.

**We note you have provided the direct URLs we requested in your manuscript, but have not updated your Data Availability statement in the system. We are just reaching out to request that you provide a new Data Availability statement incorporating those URLs, or confirm our proposed statement is an accurate, acceptable Data Availability statement：**

Please allow me to express my gratitude to the editor. Your multiple replies and reminders demonstrate the rigor and seriousness of plos one. Due to my negligence, I was unable to submit the relevant links and statements of the database, which caused a lot of inconvenience to your work. I hope to receive your forgiveness.

The relevant dataset declaration has been submitted to the system in accordance with the requirements of the “Data Availability statement”. Thank you again for your support of this article and for your patience and understanding.

With warm regards,

Shouyu Wang

wangshouyu666@126.com
